# Supplementary material for: Urinary MicroRNAs as Potential Markers for Non-Invasive Diagnosis of Bladder Cancer
Source: Int J Mol Sci. 2020 May 27;21(11):3814. doi: 10.3390/ijms21113814 (PMC7312501; doi:10.3390/ijms21113814)
Supplement: Supplementary file 1 [file ijms-21-03814-s001.pdf]

International Journal of *Molecular Sciences*

MDPI

Table S1: Overview of selected studies on the BCa-associated diagnostic and prognostic value of urinary miRNA levels

Reference	miRNAs	Issue of Analysis	Sample Type	BCa/Con	Method	Main Results
[29]	miR-125b<br>miR-183	miRNA profiling in urine for BCa marker identification (many diagn. miRs identified, e.g. miR-125b & -183, finally others better)	urine sediment	1: 30/10<br>2:151/126	1: qPCR-array<br>2: qPCR	detection of BCa e.g. by miR-125b ↓ & miR-183 ↑ (AUC: 0.726/0.722), miR-125b as prognostic marker in combination with miR-92a
[30]	miR-21<br>miR-205<br>miR-221	detection of BCa recurrence in patients under surveillance by a miRNA signature including miR-21, -205 and -221	whole urine	1: 60/21<br>2: 25/25	1: qPCR<br>2: qPCR	detection of recurrent BCa (AUC: 0.85/0.74) by a 6-miR-combination (miR-16/-21/-34a/-200c/-205/-221)
[32]	miR-125b	miRNA profiling in urine for BCa marker identification (including miR-125b & -99a)	cell-free urine	1: 6/3<br>2: 50/21	1: microarray<br>2: qPCR	detection of BCa by miR-125b ↓ (AUC: 0.813), in combination with miR-99a (AUC: 0.876), miR-125b ↓ with G ↑ (AUC: 0.819)
[33]	miR-125b<br>miR-126	detection of BCa by miRNAs (miR-125b & -126) in comparison with VUC	urine sediment	1: 8/5<br>2: 8/3	1: qPCR<br>2: qPCR	detection of BCa by miR-125b ↓ & miR-126 ↑, good decision-tree model better performing than VUC
[34]	miR-125b	miRNA profiling in urine for BCa marker identification including miR-125b	cell-free urine	1: 46/13<br>2: 27/23	1: qPCR-array<br>2: qPCR	detection of BCa by miR-125b ↓, high negative FC in all cohorts (validation AUC: 0.801), combination of miR-125b/204/5323/99a (AUC: 0.836)
[36]	miR-145	detection of BCa by miRNAs (miR-145 & -200a)	cell-free urine	207/144	qPCR	detection of BCa by miR-145 ↓, of NMIBC/ MIBC (AUC: 0.729/0.790), miR-145 ↓ in MIBC & HG-BCa
[37]	miR-145	detection of BCa by an NLR-related miRNA signature including miR-145	urine sediment	31/28	qPCR	no significant differences for miR-145 btw. BCa & controls, no association with pathol. factors
[41]	miR-96<br>miR-183	detection of UC by miRNAs (miR-96 & -183) in comparison with VUC	urine sediment	100/74	qPCR	detection of BCa by miR-96 ↑ & miR-183 ↑ (AUC: 0.831/0.817), expression ↑ with G ↑ & pT ↑
[42]	miR-96	detection of BCa by miR-96 in comparison with VUC	urine sediment	94/90	qPCR	detection of BCa by miR-96 ↑ (AUC: 0.822), not associated with pathol. factors, but with bilharziasis
[43]	miR-96<br>miR-210	HYAL1-regulating RNAs (miR-96 & -210) as BCa markers in urine	urine sediment	94/116	qPCR	detection of BCa by miR-96 ↑ & miR-210 ↑ (AUC: 0.864/0.874), associated with age, pT & bilharziasis
[44]	miR-96	miRNA profiling in urine for BCa marker identification (miR-21/-96/-126/-145/-221 …)	urine sediment	1: 27/58<br>2: 61/60	1: qPCR-array<br>2: qPCR	detection of BCa (AUC: 0.982/0.958/0.902) by 25/20/ 10 miR-combination all only including miR-96
[45]	miR-205	miRNA profiling in urine for BCa marker identification	cell-free urine	1: 66/48<br>2: 112/65	1: NGS<br>2: qPCR	amongst others miR-205 upregulated in NMIBC & MIBC vs. controls, miR-21 & -183 altered too, but none was included in final diagnostic combination
[46]	miR-126	miRNA profiling in urine for BCa marker identification (miR-126/-152/-182/-199a)	whole urine	1: 18/18<br>2: 29/18	1: qPCR-array<br>2: qPCR	detection of BCa by miR-126 ↑ (AUC: 0.747), comparable with miR-182 (AUC: 0.753)

[47]	miR-210	detection of BCa by miRNAs (miR-210, -221 & others) in relation to VUC & bilharziasis	urine sediment	188/180	qPCR-array	detection of BCa by miR-210 ↑ (AUC: 0.836), better in combination with other miRs & VUC, no association with pathol. factors & bilharziasis
[48]	miR-21	miRNA profiling in urine for BCa marker identification including miR-21	cell-free urine	1: pools<br>2: 85/45	1: qPCR-array<br>2: qPCR	high FC for miR-21, but finally detection of BCa by a 4-miR-combination without miR-21 (AUC: 0.858)
[49]	miR-21	detection of BCa by exosomal miRNAs (miR-15/-21/-31/-132/-155) in relation to VUC	urine exosomes	1: 6/3<br>2:36/24	1: microarray<br>2: qPCR	detection of BCa (AUC: 0.900) & of NMIBC (AUC: 0.912) by miR-21 ↑ & at negative VUC (AUC: 0.920)
[50]	miR-205	detection of BCa by an EMT-related miRNA signature including miR-205	cell-free urine	32/177	qPCR	detection of BCa by miR-205 ↓ (AUC: 0.845) & of NMIBC (AUC: 0.844)
[51]	miR-205	detection of BCa by an EMT-related miRNA signature including miR-205	urine sediment/ cell-free urine	51/24	qPCR	miR-205 not differentially expressed neither in urine sediment nor in cell-free urine supernatant
[52]	miR-96<br>miR-125b<br>miR-145<br>miR-205	detection of BCa by a miRNA signature in relation to VUC (miR-96/-99a/-125b/-133a/ -141/-145/-200c/-205)	not specified	30/28 + 47 w. atypical urothelial cells	qPCR	BCa-related increase of miR-200c/-205 & decrease of miR-99a/-125b/-133a/-145, miR-96 & -141 not BCa-related, only miR-99a altered in atypical cells

1 – test / screening cohort; 2 – discovery / validation cohort; AUC – area under the curve; BCa – bladder cancer; Con – control; EMT – epithelial-mesenchymal transition; FC – fold change; G – tumor grade; HG – high-grade; HYAL1 – Hyaluronidase-1; MIBC – muscle-invasive bladder cancer; NGS – next-generation sequencing; NLR – NOD-like receptors; NMBIC – non-muscle-invasive bladder cancer; pT – pathological tumor stage; qPCR – quantitative PCR; UC – urothelial cancer (including BCa, renal pelvic & ureter UC); VUC – voided urine cytology.
